# Tuberculosis: Cliché or Outsider?

**DOI:** 10.7759/cureus.53956

**Published:** 2024-02-10

**Authors:** Leonor Gama, Josiana Duarte, Inês Martins, Ana Santos e Silva, Henrique Rita

**Affiliations:** 1 Internal Medicine, Hospital do Litoral Alentejano, Santiago do Cacém, PRT; 2 Intensive Medicine Service, Hospital do Litoral Alentejano, Santiago do Cacém, PRT

**Keywords:** pulmonary tuberculosis, central nervous system tuberculosis, disseminated miliary tuberculosis, cerebral tuberculomas, antibacillary

## Abstract

Tuberculosis is an infectious disease with the potential for multisystemic dissemination, including the central nervous system (CNS). It is difficult to diagnose when the central nervous system is involved. Brain biopsy is the diagnostic method par excellence for diagnostic confirmation; however, as it is an invasive method and therefore not free from risks, before carrying it out, extra-CNS sites should be privileged, whenever available, through mycobacteriological culture.

Here, we present a case of a 34-year-old female with chronic onset of neurologic semiology, whose diagnostic evolution culminated in the diagnosis of cerebral tuberculomas and miliary tuberculosis. Rapid commencement of antibacillaty therapy led to the resolution of the neurologic deficits. Although we face a cliché clinical presentation, in the sense that is very common, the authors consider it outsider because such a presentation is rarely seen in Portugal.

## Introduction

In humans, tuberculosis (TB) is a potential multisystemic infectious disease caused by different species of mycobacteria, usually *Mycobacterium tuberculosis *[1]. Disseminated tuberculosis, an infection spread via the hematogenous route, can lead to the presence of lesions in several organs or systems, which can be manifest through different clinical manifestations [2]. It is rare in immunocompetent patients, mainly affecting patients with human immunodeficiency virus (HIV) infection, those receiving immunosuppressive therapy, elderly people, and patients with diabetesmellitusand/or alcoholism [3].

Disseminated tuberculosis with the involvement of the central nervous system (CNS) is an even rarer and more difficult diagnosis [4,5]. This represents 5-15% of extrapulmonary forms and is recognized as having high mortality [6].

Its presumptive diagnosis is based on clinical and epidemiological brightness thresholds, with brain biopsy being the method par excellence for confirming diagnostics. However, as an invasive procedure with several risks, prior to carrying it out, extra-CNS sites should be privileged whenever available, using mycobacteriological culture or molecular biologic methods to identify the pathogen [7].

## Case presentation

**Figure 1:**
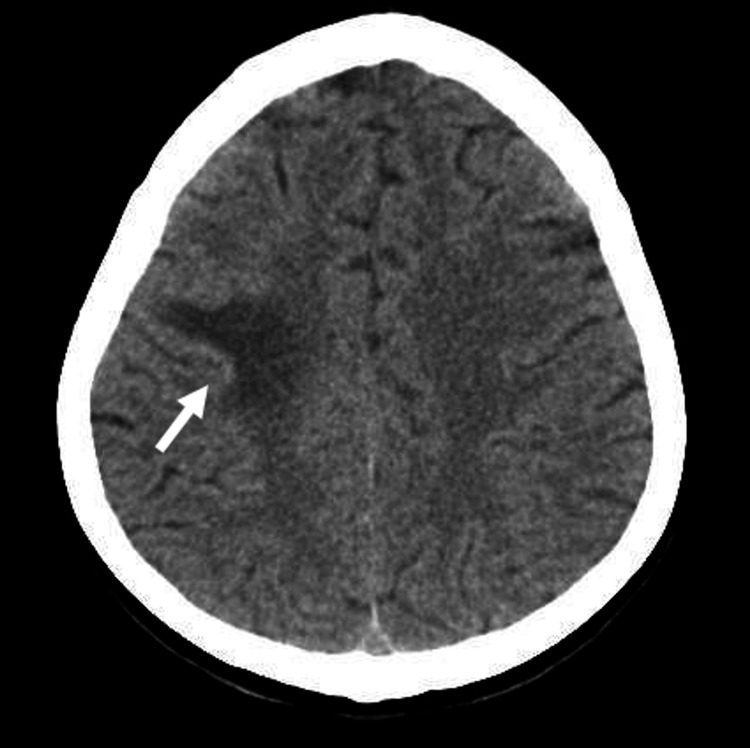
CECT demonstrates predominantly right posterior frontal subcortical hypodensity. CECT: cranioencephalic computed tomography

We present a 34-year-old female patient, originally from Nepal, but living in Portugal for five years with no travels in this period, working at a textile factory, with unknown personal history, without standard medication, and with unknown vaccination status. In March 2023, the patient attended the emergency department (ED) on several occasions due to a self-notion of decreased sensibility of the left body on a recurrent basis, but on each occasion, she was discharged for further evaluation by her attending physician which never occurred. The patient once again returned to the ED, presenting with left hemihypoaesthesia for pain that lasted for two hours. Upon admission, a fever of 39.4ºC was also observed and rhonchi on lung auscultation. No night sweats or loss of weight was detected. To better clarify the situation, considering the presence of neurologic signs with fever, we performed a cranioencephalic computed tomography (CECT) that showed a "predominant right posterior frontal subcortical hypodensity," which induces a reduction in the permeability of the loco regional cortical sulk, and which seems to translate into vasogenic edema (Figure 1).

**Figure 2:**
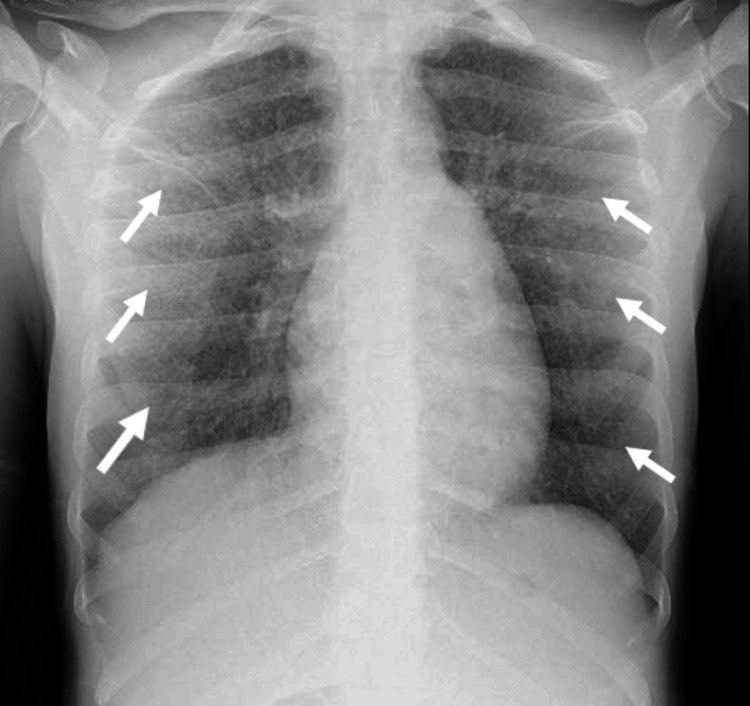
Chest X-ray showing miliary pattern.

**Figure 3:**
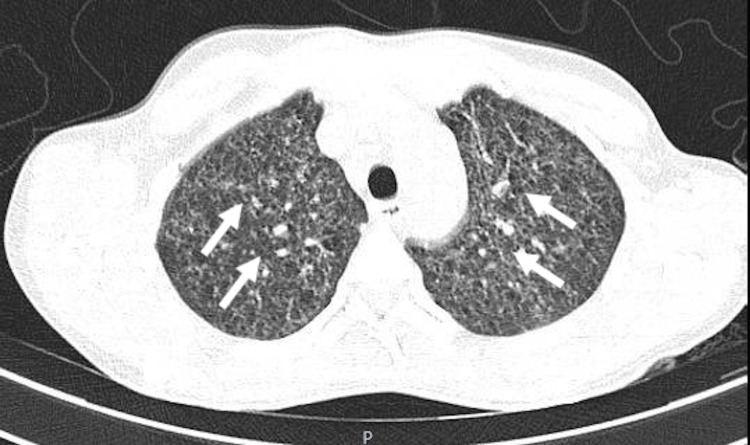
Chest CT image with micronodular pattern suggestive of infection with granulomatous agents.

Owing to fever and the abnormality in pulmonary auscultation to exclude an infectious focus (other than CNS), a chest X-ray was performed which showed a miliary pattern not evident on previous X-rays recorded less than five days before admission, also in an emergency context. To clarify the pattern visible on the chest cephalogram (Figure 2), thoracic computed tomography (CT) was performed, which showed a micronodular pattern of miliary distribution suspicious for infection with granulomatous agents (Figure 3).

At an analytical level, hyponatremia of 129 mmol/L was evident which happens in infections due to mycobacteria. However, no changes were observed in acute-phase inflammation - namely, no leucocytosis nor neutrophilia was noted and C-reactive protein measured 0.74 mg/dL (cut-off <0.50 mg/dL). Summary urine test showed no changes. Erythrocyte sedimentation rate was elevated at 70 mm (cut-off 0-10 mm). Angiotensin conversion enzyme was normal. Causes of immunosuppression, namely HIV infection, hepatitis B, hepatitis C, and syphilis were investigated and discarded. Due to the intracranial expansible lesion, we preferred not to perform a lumbar puncture and wait for the magnetic resonance imaging. Bacilloscopy was negative and blood cultures were performed without any isolation for bacteria or fungi.

Therefore, we opted for hospitalization with the diagnostic hypothesis of miliary tuberculosis and space-occupying brain lesion with unknown etiology, being more likely, and given the clinical, epidemiological context and PCR blood test for *Mycobacterium tuberculosis* positive, of tuberculous etiology.

Owing to the high clinical suspicion, empirical therapy was initiated with antibacterial drugs (isoniazid at a dose of 5 mg/kg, rifampicin 10 mg/kg, pyrazinamide 25 mg/kg, and ethambutol 20 mg/kg). We chose this treatment because it is recommended by the World Health Organization (WHO).

**Figure 4:**
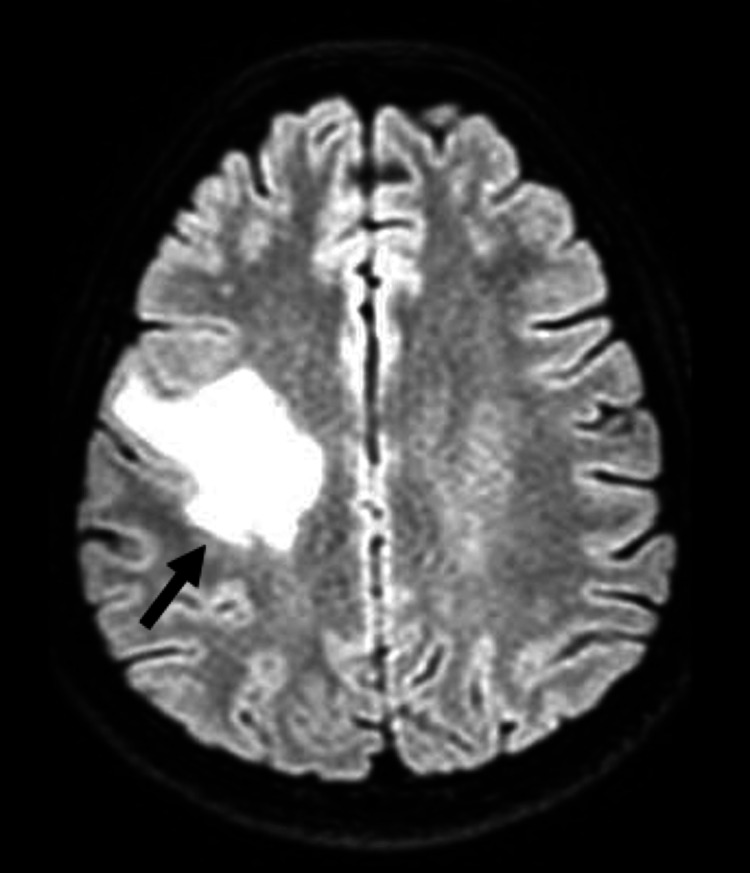
Contrast-enhanced MRI with evidence of lesions suggestive of cerebral tuberculomas (T2-weighted imaging).

**Figure 5:**
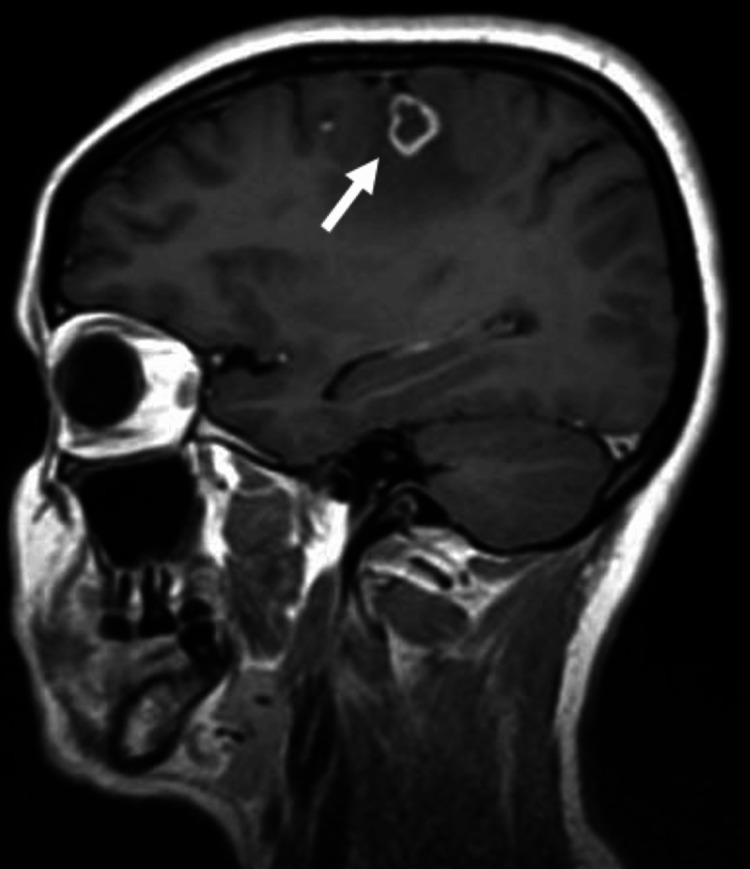
Contrast-enhanced MRI with evidence of lesions suggestive of cerebral tuberculomas (T2-weighted imaging).

**Figure 6:**
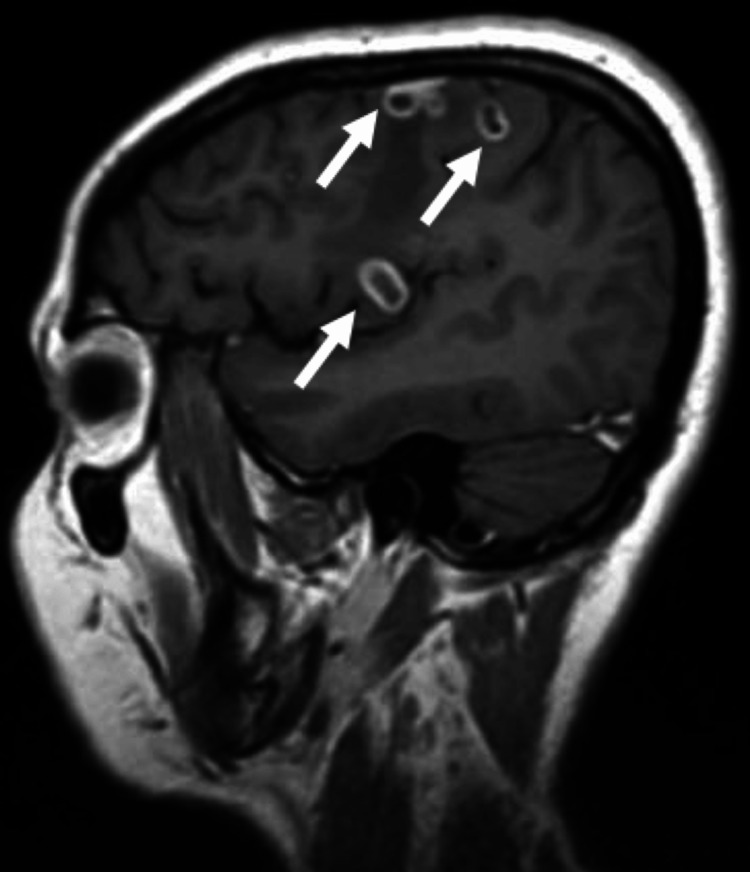
Contrast-enhanced with evidence of lesions suggestive of cerebral tuberculomas (T2-weighted imaging).

During hospitalization, we performed brain contrast-enhanced magnetic resonance imaging to better clarify the space-occupying brain (Figures 4-6). From the results of the imaging examination, several ring lesions with hyper signal on the periphery on T2-weighted imaging, and in the central area more hypointense on T2, namely in the left temporal regions, with larger dimensions posterior to the right, it appears that there are small contiguous cutaneous lesions in the right frontoparietal region with moderate perilesional edema and we also observed some areas of hemorrhage with cortico-pial hemosiderin. We noted another small lesion on the left internal occipital ring. The injuries do not demonstrate a significant regional mass effect. These aspects are compatible with multiple tuberculomas in the context of cerebral tuberculosis. Due to an infection of CNS, we also initiated prednisolone 40 mg/day for four weeks and then we started lowering doses, as WHO recommends.

As the investigation of injuries was not without risk and once there was evidence of extracerebral infection, bronchoscopy with bronchoalveolar lavage was performed, from which *Mycobacterium tuberculosis -* the causative agent of tuberculosis - was isolated. 

The patient was discharged with improvement in neurological deficits and rehabilitation, with 30 days of antibacterial therapy and assured social context. Additionally, she was referred to the Pneumological Diagnostic Center and the Internal Medicine appointment, for continued treatment and surveillance for 12 months.

## Discussion

The clinical features of tuberculosis are usually non-specific and may present with fever, weight loss, night sweats, anorexia, and weakness. The physical findings in descending order are fever, wasting, hepatomegaly, pulmonary findings, lymphadenopathy, and splenomegaly [6]. In our case, only fever and pulmonary findings were observed when the patient was admitted to the ER.

In this case, the presumptive diagnosis of tuberculomas, in the context of disseminated tuberculosis, was strongly indicated by the patient's origin from a country where TB is endemic and her neurologic symptoms. Disseminated tuberculosis with involvement of the central nervous system represents 5-15% of extrapulmonary forms and is recognized as having high mortality [7]. The involvement of the central nervous system can be manifested diffusely as tuberculous meningitis, localized as tuberculoma or tuberculous abscess, or in extradural and intradural spinal infections [8]. Tuberculomas are clearly uncommon in the Western world, but in endemic countries represent 20-30% of all intracranial tumors [9]. As a result of multilateral migration and globalization in times of humanitarian crises, Western countries face a possible increase in the incidence of central nervous system tuberculosis [8].

However, it can affect almost any part of the body. Tubercular lymphadenitis is the most common form of extrapulmonary TB [10]. There are about 800 lymph nodes in the body of a healthy adult human being. Three hundred of them are in the head and neck area [11]. To choose the right antibiotic therapy, it is necessary to know the etiological bacterial spectrum of this purulent disease [12]. Cervical lymphadenopathy (92.6%) was the most common presentation of peripheral tuberculous lymphadenopathy [10]. Suppurative lymphadenitis occurs in patients with reduced congenital or acquired immune forces, and the main causative agents of the disease are the Gram-positive bacteria *Staphylococcus aureus*, *Streptococcus pyogenes*, and *Mycobacterium tuberculosis *[13]. Nevertheless, the patient never presented this symptom. 

As soon as we initiated the treatment, we observed a clinical improvement of left hemihypoaesthesia to pain. After one week of treatment, she described that she could feel the left hemibody again and without a fever. In two weeks, we observed the total resolution of pulmonary findings on X-ray. She was discharged after 30 days of treatment with no complaints.

So, it's very important to initiate the treatment as soon as we have clinical suspicion because the mortality rate without specific treatment can be as high as 100% if not timely diagnosed. It is important to think about all the possibilities of diagnosis and initiate the treatment as soon as the possibility of tuberculomas is made to prevent more complications.

## Conclusions

Owing to their rarity, non-specific symptoms, such as left hemihypoaesthesia for pain, and equivocal CT findings, intracranial tuberculomas remain a clinical challenge. This study required extensive research of the etiology of the symptoms and clinical signs of the disease because the patient didn't have the typical symptoms of night sweats, or weight loss, or lymphadenomegaly. In endemic countries, these forms of tuberculosis continue to be reported frequently. In essence, it's a cliché clinical presentation because it's still so common, but it can also be viewed as an outsider because in Portugal it's a rare disease.
